# Terahertz in-line digital holography of human hepatocellular carcinoma tissue

**DOI:** 10.1038/srep08445

**Published:** 2015-02-13

**Authors:** Lu Rong, Tatiana Latychevskaia, Chunhai Chen, Dayong Wang, Zhengping Yu, Xun Zhou, Zeyu Li, Haochong Huang, Yunxin Wang, Zhou Zhou

**Affiliations:** 1 College of Applied Sciences & Beijing Engineering Research Center of Precision Measurement Technology and Instrument, Beijing University of Technology, No. 100 Pingleyuan Rd., Beijing, 100124, China; 2 Physics Department, University of Zurich, Winterthurerstrasse 190, 8057 Zurich, Switzerland; 3 Research Centre of Laser Fusion, CAEP, Mianyang, 621900, China; 4 Terahertz Research Centre, CAEP, Mianyang, 621900, China; 5 Department of Occupational Health, Third Military Medical University, Chongqing, 400038, China

## Abstract

Terahertz waves provide a better contrast in imaging soft biomedical tissues than X-rays, and unlike X-rays, they cause no ionisation damage, making them a good option for biomedical imaging. Terahertz absorption imaging has conventionally been used for cancer diagnosis. However, the absorption properties of a cancerous sample are influenced by two opposing factors: an increase in absorption due to a higher degree of hydration and a decrease in absorption due to structural changes. It is therefore difficult to diagnose cancer from an absorption image. Phase imaging can thus be critical for diagnostics. We demonstrate imaging of the absorption and phase-shift distributions of 3.2 mm × 2.3 mm × 30-μm-thick human hepatocellular carcinoma tissue by continuous-wave terahertz digital in-line holography. The acquisition time of a few seconds for a single in-line hologram is much shorter than that of other terahertz diagnostic techniques, and future detectors will allow acquisition of meaningful holograms without sample dehydration. The resolution of the reconstructions was enhanced by sub-pixel shifting and extrapolation. Another advantage of this technique is its relaxed minimal sample size limitation. The fibrosis indicated in the phase distribution demonstrates the potential of terahertz holographic imaging to obtain a more objective, early diagnosis of cancer.

On the electromagnetic spectrum, terahertz waves are between infrared radiation and microwaves, and they share some of the properties of each of these spectra. Similar to infrared radiation, terahertz waves are sensitive to infrared-active modes and provide a better contrast in the imaging of soft biomedical tissues than do X-rays. Similar to microwave radiation, and in contrast to X-rays, terahertz radiation can penetrate non-conducting materials, such as clothes, without causing ionising radiation damage. These properties make terahertz waves suitable for non-invasive biomedical imaging and cancer diagnosis^1^,^2^,^3^,^4^,^5^,^6^,^7^,^8^,^9^,^10^,^11^,^12^,^13^.

For most cancers, confident diagnosis at an early stage is a challenging task, and several cross-checking tests are typically performed. Diseased or cancerous tissues contain more interstitial water than healthy tissues as a result of oedema or increased vascularity^3^, and the analysis of water content can be performed by measuring the absorption of terahertz radiation^1^,^2^,^3^,^4^,^5^,^6^,^7^,^8^,^9^,^10^,^11^,^12^. However, the absorption contrast is affected not only by the water content but also by the structural changes caused by cancer^4^. Intentionally dehydrated cancerous tissue or massively inflamed liver tissue has a lower absorption than normal tissue at certain terahertz frequencies^5^,^6^. Thus, there are two competing influences on the absorption of cancerous tissues: an increase in absorption due to higher water content^3^ and a decrease in absorption due to the altered cell density and protein content^2^. It is therefore difficult to evaluate the degree of cancerous damage from absorption imaging alone, and phase-shift imaging can be critical for diagnostics.

Terahertz time domain spectroscopy (THz-TDS)^14^,^15^ is capable of extracting both the amplitude and phase information of the pulsed terahertz signal, enabling the absorption coefficient and the refractive index of the object to be obtained simultaneously^3^,^4^,^5^. To further determine the histological features of the tissue, the image is mapped out pixel by pixel by raster scanning the object based on THz-TDS in the reflective mode^6^,^7^,^8^,^9^, also referred to as terahertz pulsed imaging (TPI)^16^. However, TPI has the following disadvantages: the sample size must be larger than 5 mm × 5 mm to avoid diffraction effects at the sample's edges, and dehydration must be prevented during the slow acquisition time, which varies from 10 min for a 26 mm × 20 mm sample^6^ to 6 hours for an 18.7 mm × 18.7 mm^9^ sample depending on the hardware and the scanning step. Continuous-wave (CW) terahertz imaging of cancerous tissues has become more popular due to the ready availability of commercial high-power terahertz laser systems. However, the acquisition time of the CW terahertz fibre-scanning near-field imaging technique is even longer than that of TPI and is approximately 50 min for 10 mm × 10 mm samples^10^,^11^,^12^.

Holography^17^, by contrast, allows recording of both the amplitude and the phase distribution of the scattered waves in a single holographic record, which can then be used to retrieve the absorption and phase-shifting properties of the object by numerical reconstruction^18^. Terahertz digital holography^13^,^19^,^20^,^21^,^22^,^23^ has been applied to achieve the simultaneous reconstruction of absorption and phase-shift distributions^13^,^21^, and a resolution of approximately 0.2 mm was achieved^23^, although this technique has not been applied to image cancer tissues.

## Results

### Experimental setup

**Figure 1:**
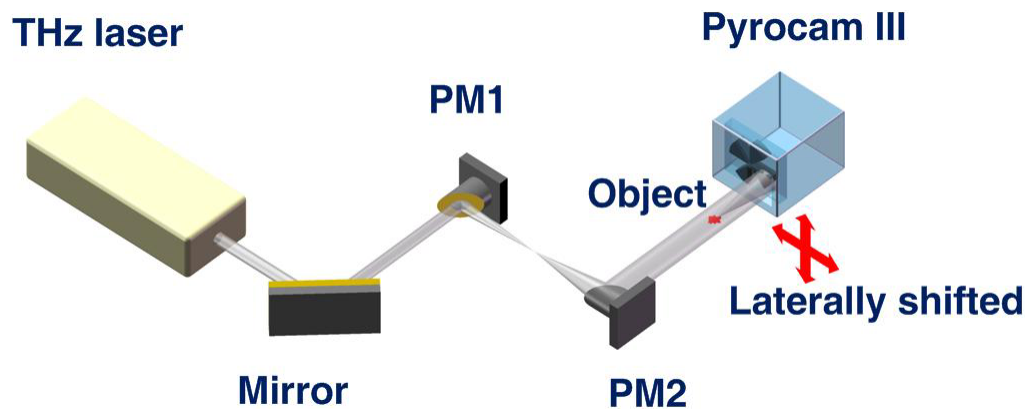
Schematic layout of the setup for continuous-wave terahertz in-line digital holography. An output terahertz laser beam of 11 mm in diameter was expanded to 22 mm in diameter by using two gold-coated off-axis parabolic mirrors, PM1 and PM2, with effective focal lengths of 76.2 mm and 152.4 mm, respectively.

In the present study, we imaged cancerous tissues using in-line holography. A Gabor-type in-line holographic scheme^17^ does not require optical elements between the sample and the detector, which allows aberrations to be avoided and makes this approach a good choice for terahertz imaging^13^,^23^. An experimental set-up for terahertz in-line digital holography is depicted in Fig. 1. A far-infrared radiation laser system pumped by a CO_2_ laser was used to provide a 2.52 THz CW beam with a wavelength of 118.83 μm. The in-line hologram was formed by interference between the wave scattered by the object and the unscattered beam, and it was recorded by a pyroelectric array detector with 124 × 124 pixels, a pixel size of 85 μm × 85 μm, and a pitch of 100 μm × 100 μm. The conditions for realising holography have been outlined by Dennis Gabor^24^: for the successful reconstruction of an object from its in-line hologram, the area occupied by the object must be approximately 1% of the entire area illuminated by the incident wave. Thus, in in-line holography, the smaller the object size, the more reference wave information there is in the hologram and the more reliable is the object reconstruction. This condition relaxes the requirement for the minimal size of the sample. However, the sample must not be smaller than the wavelength of the probing waves.

**Figure 2:**
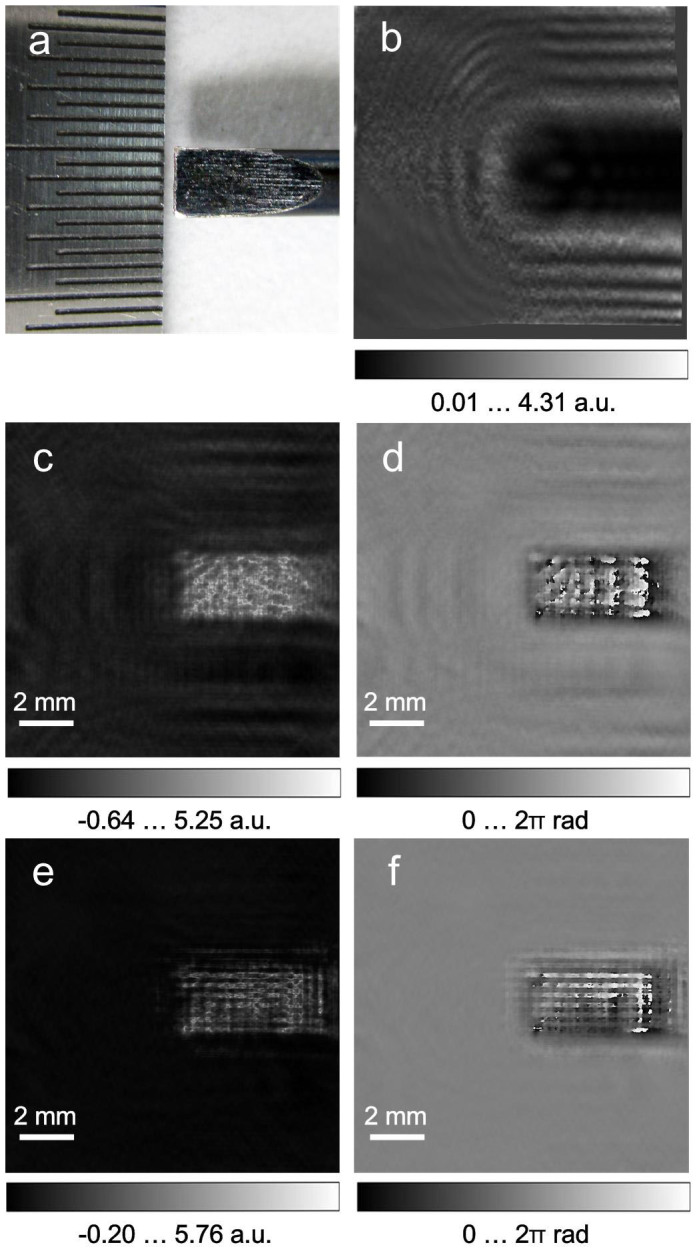
Terahertz in-line hologram of the steel blade of a screwdriver and its reconstructions. (a), photo of the metal blade. (b), normalised hologram. (c), reconstructed absorption distribution *a*(*x*,*y*). (d), reconstructed phase-shift distribution *φ*(*x*,*y*). (e–f), reconstructed absorption and phase distributions after 100 iterations, respectively.

As a calibration object to test the absorption and phase imaging of our terahertz in-line holographic setup, we selected a metallic object – a steel screwdriver blade, a photo of which is shown in Fig. 2(a). The sample was placed at a distance of 19 mm from the detector. To enable the simultaneous reconstruction of the absorption and phase distributions from a single hologram, the hologram must be normalised with respect to the background intensity^25^,^26^. Thus, images with and without the sample were recorded. To increase the sampling of the recorded images, the detector was laterally shifted in a stepwise fashion (as indicated in Fig. 1) with sub-pixel-size steps for a total of 25 steps. At each detector position, 1,000 frames of both the hologram and background images were recorded and averaged (see Methods: Statistical averaging of individual frames). The sampling required 1/48 sec per single frame and 20.83 sec per 1,000 frames; thus, the total acquisition time for all 25 steps was approximately 9 min. The resulting 25 averaged holograms and 25 background images were each upsampled from 124 × 124 pixels to 992 × 992 pixels and aligned using a sub-pixel registration method^27^. The normalised hologram (see Methods: Hologram normalisation) is shown in Fig. 2(b). The reconstructed absorption and phase distributions are shown in Fig. 2(c)–(f) (see Methods: Hologram reconstruction). The metal blade of the screwdriver is relatively thin and exhibits finite absorption of terahertz waves, and some phase contrast is also observed. Moreover, after applying an iterative reconstruction routine^25^ (see Methods: Hologram reconstruction), the twin image in the reconstructions is suppressed and the pronounced fringe structure becomes apparent in the phase distribution.

### Cancerous liver sample

A cancerous liver sample was obtained from a patient who underwent a liver resection. The section of liver was diagnosed as containing a hepatocellular carcinoma (HCC) tumour by histology. The tissue was stored immediately after resection at −80°C. While still frozen, it was cut into 30-μm-thick slices with a microtome, and the slices were placed in pH-balanced saline (pH 7.4), where they were kept for two days prior to the holographic experiments. For the holographic imaging, a patch of approximately 3.2 mm × 2.3 mm was excised from one of the slices and mounted on a 3-mm-thick quartz slide. The slide was placed approximately 25 mm in front of the detector and tilted at an angle of approximately 12° relative to the incident beam to minimise the unwanted interference caused by diffraction at the slide. The study was approved by the Ethics and Scientific Committees of the Third Military Medical University, China.

### Hologram acquisition and reconstruction

**Figure 3:**
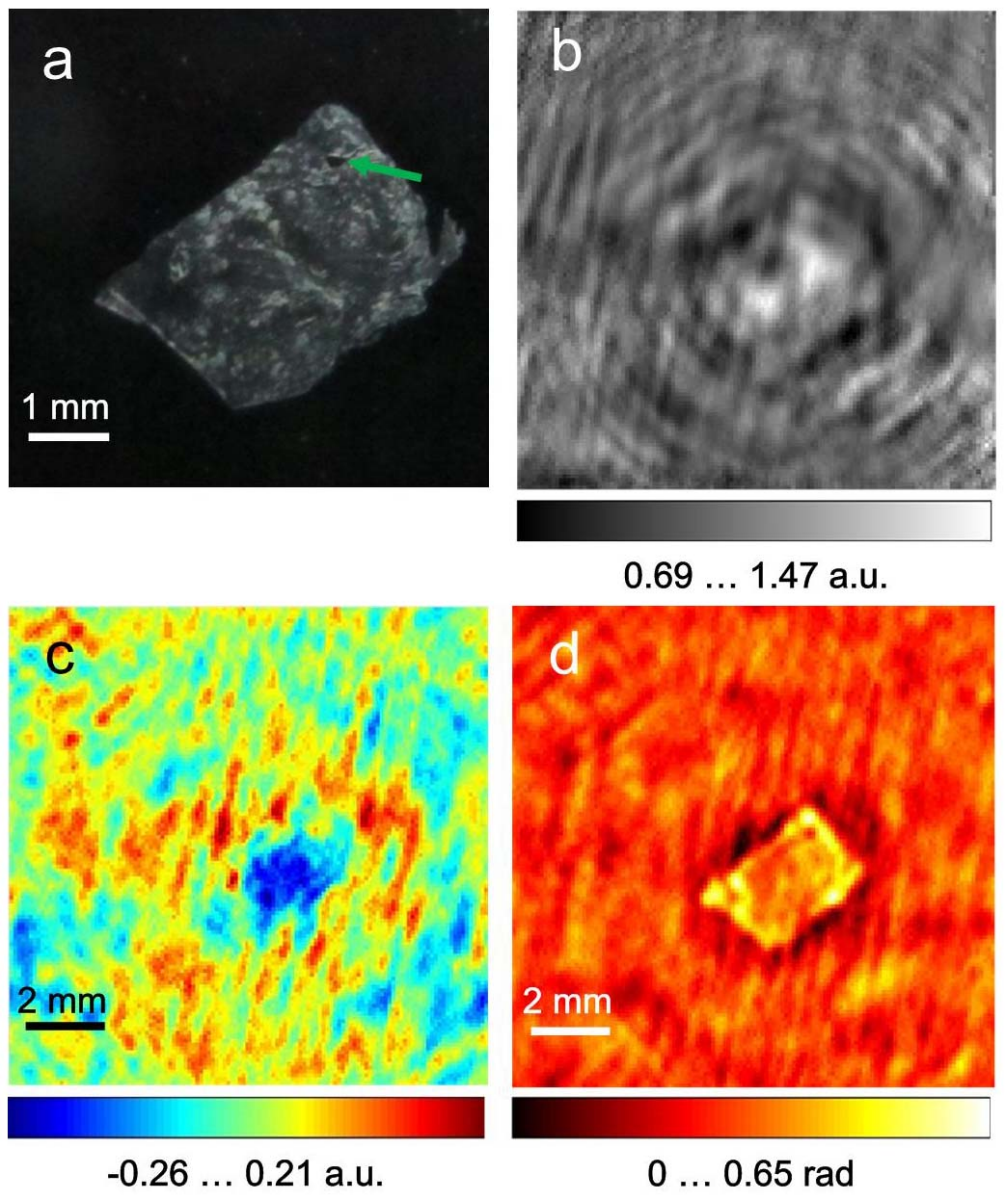
Terahertz in-line hologram of human hepatocellular carcinoma tissue and its reconstructions. (a), photo of the sample after holographic data acquisition. (b), normalised hologram obtained at a selected detector position; the hologram size is 12.4 × 12.4 mm^2^ sampled with 124 × 124 pixels. (c), reconstructed absorption distribution *a*(*x*,*y*). (d), reconstructed phase-shift distribution *φ*(*x*,*y*).

**Figure 4:**
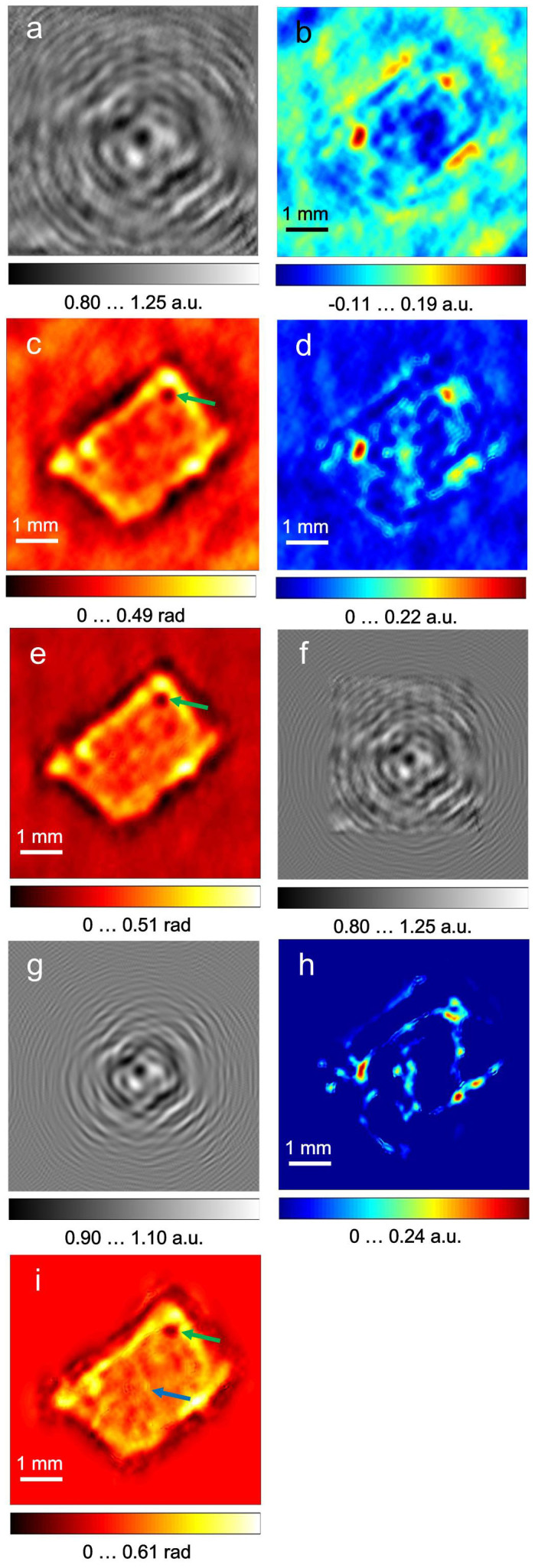
Upsampled holograms of human hepatocellular carcinoma tissue and their reconstructions. (a), normalised hologram. (b–c), object absorption and phase-shift distributions reconstructed from the normalised hologram. The absorption up to *a* = 0.22 a.u. means that up to 36.21% of the incident radiation was absorbed. (d–e), reconstructed absorption and phase-shift distributions after 200 iterations, respectively. (f), extrapolated hologram after 200 iterations. (g), the amplitude of the complex-valued field at the detector after the last iteration. (h–i), absorption and phase-shift distributions reconstructed from the extrapolated hologram.

A digital photo of the sample is shown in Fig. 3(a). A single normalised hologram 124 × 124 pixels in size, resulting from the averaging of 1,000 frames recorded at a selected detector position, is shown in Fig. 3(b). The reconstructed absorption and phase-shift distributions from the normalised hologram are shown in Fig. 3(c) and Fig. 3(d), respectively. Thus, a single hologram acquired during a 20.83-sec interval is sufficient to arrive at low-resolution reconstructions of the absorption and phase-shifting properties of the sample. To retrieve these distributions at a higher resolution, the holograms acquired at different detector positions were upsampled, aligned and averaged in the same manner as described above for the test sample. The reconstructed absorption and phase-shift distributions from the resulting normalised hologram of 992 × 992 pixels size are shown in Fig. 4(b) and Fig. 4 (c). Whereas the reconstructed absorption of the sample is quite low, which can be explained by the dehydration during data acquisition, the reconstructed phase-shift distribution demonstrates good contrast, matches well with the contour of the tissue sample, and exhibits detailed features not observed in the absorption image. For example, the hole in the top left corner of the sample (indicated by a green arrow in Fig. 3(a)), which could be either a cut across a vessel or the result of damage caused by the freezing procedure, is pronounced in the phase reconstruction shown in Fig. 4 (c).

The small numerical aperture (N.A. = 0.24) of the set-up leads to a limited resolution in the reconstructed images^28^. It is, however, possible to increase the N.A. and therefore the resolution by a numerical extrapolation of the experimental hologram^29^,^30^. The hologram was extrapolated by an iterative reconstruction based on the error-reduction algorithm^31^, wherein the propagation between the detector and the object planes was calculated using the angular spectrum approach^32^ (see Methods: Extrapolation procedure). The resulting extrapolated hologram is shown in Fig. 4(f), where the edges of the original hologram are still visible. In comparison, the amplitude of the complex-valued field at the detector after the last iteration shown in Fig. 4(g) has a smoother appearance than the extrapolated normalised hologram. The discrepancy between Fig. 4(f) and Fig. 4(g) can be explained by the fact that the extrapolated hologram is created by substantial, and therefore not artefact-free, data analysis, whereas the amplitude at the hologram plane after each iteration approaches that of an “ideal” hologram.

The reconstructed absorption and phase-shift distributions from the extrapolated hologram are shown in Fig. 4(h) and Fig. 4(i). The phase-shift distribution reconstructed from the extrapolated hologram (Fig. 4(i)) reveals additional details of the inner structure that are not observed in the reconstruction of the normalised hologram (Fig. 4(d) and Fig. 4(e)). For example, the vertical line that can be seen in the middle of the tissue (indicated by a blue arrow in Fig. 4(i)) is apparent in the reconstruction of the extrapolated hologram and can be attributed to the fibrosis in the tissue. Fibrosis is often an indication of cirrhosis, which when untreated can develop into HCC. Thus, in our sample, the phase image contains more structural information, which can be analysed for indications of cancer.

**Figure 5:**
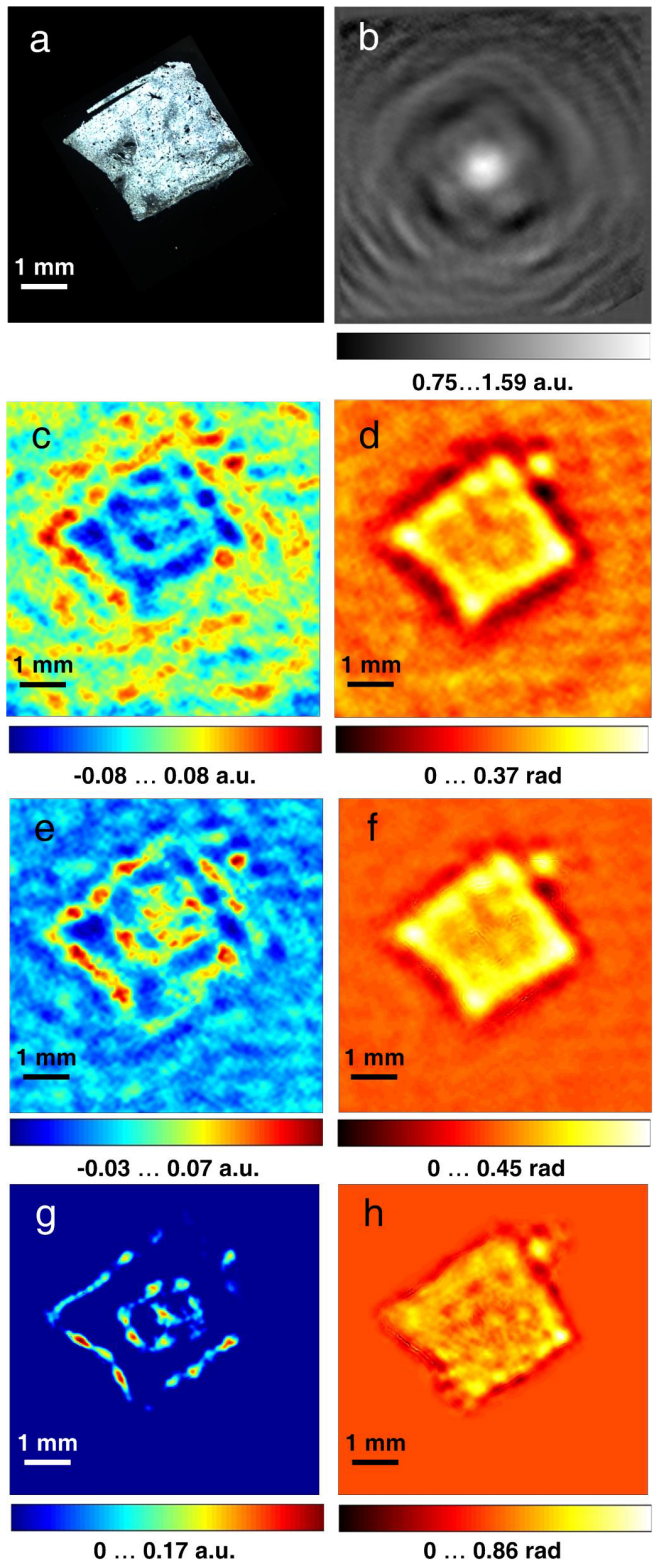
Terahertz in-line hologram of human healthy liver tissue and its reconstructions. (a), photo of the sample after holographic data acquisition. (b), normalised hologram. (c–d), object absorption and phase-shift distributions reconstructed from the normalised hologram. (e–f), absorption and phase-shift distributions reconstructed from Fig. 5(b) after 200 iterations, respectively. (g–h), absorption and phase-shift distributions reconstructed from the extrapolated hologram.

To cross-validate our results, we also performed imaging of healthy liver tissue, which was placed at a distance of 36.6 mm from the detector. The imaging and reconstruction was done in exactly the same manner as for the cancerous tissue. The results are shown in Fig. 5. The reconstructed phase-shift image of the healthy liver tissue (Fig. 5 (h)) has a more uniform appearance than that of the cancerous tissue (Fig. 4(i)).

### Estimation of resolution

Quantitatively, the increase in resolution can be estimated as follows. The intrinsic resolution of in-line holography is given by 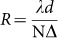
18, where *λ* is the wavelength, *d* is the distance between the sample and the detector, N is number of pixels, Δ is the pixel size, and NΔ × NΔ is the size of the hologram. For terahertz in-line holography at sample-to-detector distances greater than 3 mm, the resolution is given by the intrinsic resolution^33^. In the formula for calculating the resolution^18^, the hologram size is limited to the area where the interference pattern is observed. For the single holographic record shown in Fig. 3, as well as for the upsampled hologram shown in Fig. 4, the size of the hologram is 12.4 × 12.4 mm^2^ and the resolution is *R* = 240 μm. After extrapolation, the effective size of the hologram has been increased to 18.75 × 18.75 mm^2^ with a resolution of *R* = 158 μm.

### Acquisition time and hydration level

**Figure 6:**
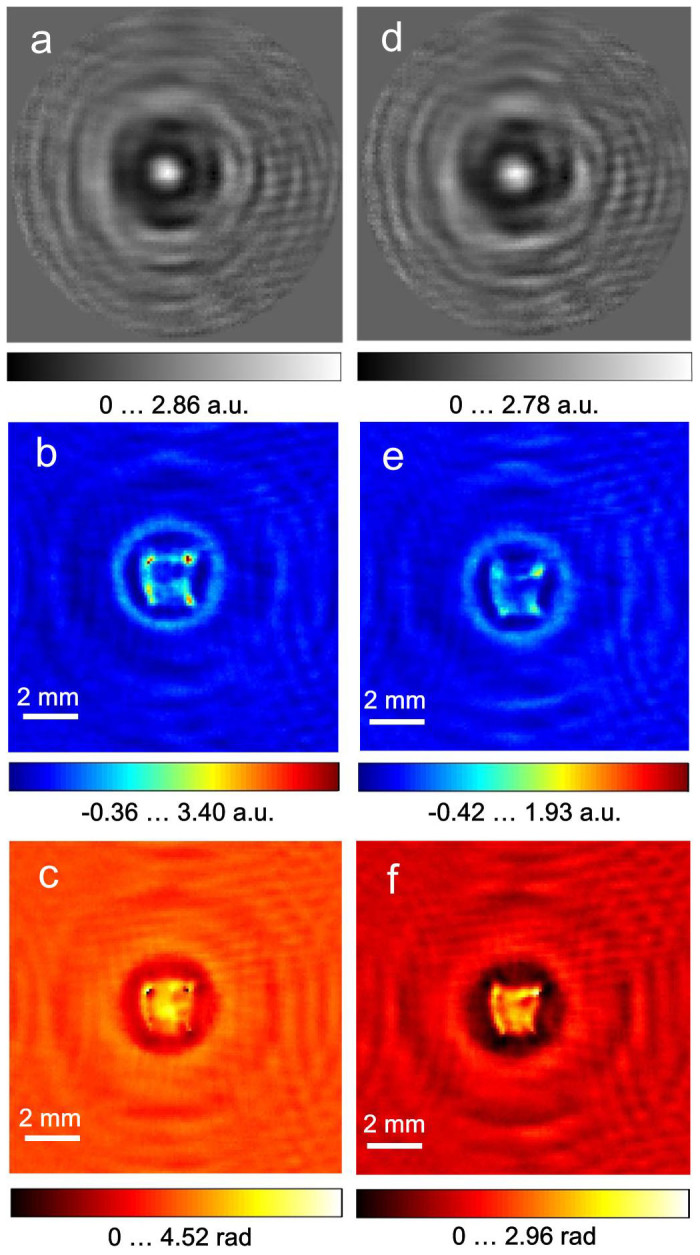
Terahertz in-line holograms of mouse healthy liver tissue and their reconstructions. (a), normalised hologram from the first 100 frames of 1,000 frames in total. (b–c), object absorption and phase-shift distributions reconstructed from Fig. 6(a). (d), normalised hologram from the last 100 frames of the 1,000 frames. (e–f), absorption and phase-shift distributions reconstructed from Fig. 6(d). The rings in the reconstructions are due to the applied round cosine-like apodisation filter, which sets the intensity values at the holograms' edges to zero, as seen in (a)and (d), to avoid reflection of the signal at the edges of the holograms during Fourier transforms.

To investigate the change in the sample hydration during the acquisition of in-line holograms as a function of acquisition time, we recorded holograms of a sample of 60-μm-thick healthy mouse liver. The sample was twice as thick as the human samples; thus, the absorption was expected to be stronger. The entire period of data acquisition was less than 1 min, including retrieval of the sample from the saline. In less than 5 min (starting from the time the sample was mounted on the slide), the samples completely rolled up. For timing reasons, we thus recorded 1,000 frames of holograms without shifting the detector. The first 100 (1–100) and the last 100 (901–1,000) frames were averaged into two holograms with good signal-to noise ratio, which were then reconstructed. The results are shown in Fig. 6. It is obvious from a comparison of Fig. 6(b) and Fig. 6(e) that the maximum absorption for the first hologram (frames 1–100) is higher than the maximum absorption for the second hologram (frames 901–1,000). This finding demonstrates that the sample quickly dehydrated during the acquisition procedure. Once a better detector with better dynamics and larger number of pixels is available, the number of frames, and therefore the acquisition time, can be further reduced. This efficiency, in turn, can enable better preservation of the hydration level of samples.

## Discussion

With the method proposed here, the absorption and the phase-shift distributions were simultaneously reconstructed from a normalised holographic record, and both distributions can contribute to the diagnosis of early-stage cancer, for example, by imaging an indication of fibrosis in the liver. The reconstructed absorption is low, which indicates that the sample is transparent to the terahertz waves and that imaging of the internal structure of the sample is possible. Thus, in terahertz in-line holography, the phase image exhibits more structure and can be used for diagnosis. Further development of terahertz array detectors with larger areas and smaller pixel sizes will eliminate the need for recording and averaging over a sequence of holograms, and a single-shot hologram will be sufficient for high-resolution reconstruction of the sample, which will ultimately reduce the acquisition time to just a few seconds and thus allow the preservation of the natural hydration level. Conventional cancer diagnosis by biopsy can be complemented by the method proposed here because the tissues extracted for biopsy have the optimal size for terahertz digital holography. Overall, we believe that these results will encourage the development of terahertz digital holography for further biomedical applications.

## Methods

### Statistical averaging of individual frames

Owing to the thermal working principle of the pyroelectric detector, zero or negative values appeared at some pixels in some frames. For each pixel, a histogram of its intensity values (bin size = 1,000^1/3^ = 10) was plotted and fitted with a Gaussian distribution function, and the mean of the Gaussian distribution function was considered to be the pixel value^13^. The pixels whose values were zero for all 1,000 frames were marked as “dead” pixels; they were set free of constraints during the phase retrieval, which allowed their values to be retrieved.

### Hologram normalisation

The averaged hologram *H_0_* was divided by the averaged background *B_0_* to obtain the normalised hologram. Owing to the inhomogeneous absorption of the quartz slide and the slightly unstable intensity of the terahertz beam, the intensity of *H_0_/B_0_* did not vary from a value of approximately 1 at the edges of the hologram, where the amplitude of the object wave was very weak and only the reference wave was detected. To solve this problem, *H_0_/B_0_* was smoothed 1,000 times using a low-pass filter, which gave *B′*, and was then divided by *B′* to give *H*_Normalised_ *= H_0_/(B_0_B′).* Fourier domain filtering was applied to eliminate the horizontal fringes created by the detector scanning procedure.

### Hologram reconstruction

The complex-valued transmission function of the sample is described by the function *t*(*x*,*y*) = exp[−*a*(*x*,*y*)−*iφ*(*x*,*y*)], where *a*(*x*,*y*) is the absorption and *φ*(*x*,*y*) is the phase shift introduced by the object in the passing wave^25^. The transmission function of the sample *t*(*x*,*y*) was reconstructed by the propagation of the optical field from the detector plane backwards to the object plane using the angular spectrum approach^32^:
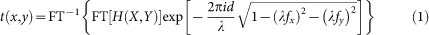
where FT and FT^−1^ are the Fourier transform and inverse Fourier transform, respectively, and *f*_x_ and *f*_y_ are the spatial frequencies. The distribution of the reconstructed transmission function was refined by applying iterative reconstruction^25^, where the constraint of positive absorption values was applied.

### Extrapolation procedure

Extrapolation is similar to the procedure explained elsewhere^29^. For the first iteration, the complex-valued distribution in the recording plane was created as follows. The amplitude was obtained by padding the square root of the normalised hologram *H*_Normalised_ (size of 992 × 992 pixels) up to a size of 1,500 × 1,500 pixels with noise. The phase distribution was assumed to be randomly distributed in the range [−π, π]. The phase retrieval procedure was based on the error-reduction algorithm^31^ applied for 200 iterations, and the field propagation between the detector and the object plane was calculated by using the angular spectrum method^18^. The following constraints were applied in the detector plane: the amplitude of the central 992 × 992 pixel region was replaced by the square root of the hologram. The amplitude values at the remaining pixels and the phase values at all pixels were updated after each iteration. In the object domain, the constraints of a positive absorption^25^ and a supporting mask were applied.

## Author Contributions

L.R., X.Z. and D.W. designed the experiments; Z.Y., C.C. and Z.Z. prepared the samples; H.H., Z.L. and Y.W. performed the experiments; T.L. developed the numerical routines and performed the reconstructions; and L.R., T.L. and C.C. analysed the reconstructions and wrote the manuscript.

## References

[b1] Yin X., Ng B. & Abbott D. Terahertz Imaging for Biomedical Applications: Pattern Recognition and Tomographic Reconstruction. (Springer, New York, 2012).

[b2] Park G. *et al.* Convergence of Terahertz Sciences in Biomedical Systems. (Springer, Dordrecht, 2012).

[b3] Png G. M. *et al.* The impact of hydration changes in fresh bio-tissue on THz spectroscopic measurements. Phys. Med. Biol. 53, 3501–3517 (2008). 18552421 10.1088/0031-9155/53/13/007

[b4] Sy S. *et al.* Terahertz spectroscopy of liver cirrhosis: investigating the origin of contrast. Phys. Med. Biol. 55, 7587–7596 (2010). 21098916 10.1088/0031-9155/55/24/013

[b5] Nishizawa J. *et al.* THz imaging of nucleobases and cancerous tissue using a GaP THz-wave generator. Opt. Commun. 244, 469–474 (2005).

[b6] Zhang C. H. *et al.* Terahertz imaging on subcutaneous tissues and liver inflamed by liver cancer cells. THz Sci. Tech. 5, 114–123 (2012).

[b7] Woodward R. M. *et al.* Terahertz pulse imaging of ex vivo basal cell carcinoma. J. Invest Dermatol. 120, 72–78 (2003). 12535200 10.1046/j.1523-1747.2003.12013.x

[b8] Yu C., Fan S., Sun Y. & Pickwell-MacPherson E. The potential of terahertz imaging for cancer diagnosis: A review of investigations to date. Quant. Imaging Med. Surg. 2, 33–45 (2012). 23256057 10.3978/j.issn.2223-4292.2012.01.04 PMC3496499

[b9] Formanek F., Brun M. & Yasuda A. Contrast improvement of terahertz images of thin histopathologic sections. Biomed. Opt. Express 2, 58–64 (2011). 21326635 10.1364/BOE.2.000058 PMC3028498

[b10] Chiu C. *et al.* All-terahertz fiber-scanning near-field microscopy. Opt. Lett. 34, 1084–1086 (2009). 19340227 10.1364/ol.34.001084

[b11] Chen H. *et al.* Performance of THz fiber-scanning near-field microscopy to diagnose breast tumors. Opt. Express 19, 19523–19531 (2011). 21996893 10.1364/OE.19.019523

[b12] Chen H. *et al.* The diagnosis of human liver cancer by using THz fiber-scanning near-field imaging. Chin. Phys. Lett. 30, 030702 (2013).

[b13] Rong L. *et al.* Terahertz in-line digital holography of dragonfly hindwing: amplitude and phase reconstruction at enhanced resolution by extrapolation. Opt. Express 22, 17236–17245 (2014). 25090537 10.1364/OE.22.017236

[b14] Smith P. R., Auston D. H. & Nuss M. C. Subpicosecond photoconducting dipole antennas. IEEE Quantum Electronics 24, 255–260 (1988).

[b15] Grischkowsky D., Keiding S., van Exten M.& Fattinger C. Far-infrared time domain spectroscopy with terahertz beams of dielectrics and semiconductors. J. Opt. Soc. Am. B 7, 2006–2015 (1990).

[b16] Hu B. & Nuss M. Imaging with terahertz waves. Opt. Lett. 20, 1716–1718 (1995). 19862134 10.1364/ol.20.001716

[b17] Gabor D. A new microscopic principle. Nature 161, 777–778 (1948). 18860291 10.1038/161777a0

[b18] Schnars U. & Jüptner W. Digital Holography: Digital Hologram Recording, Numerical Reconstruction, and Related Techniques. (Springer, Berlin, 2004).

[b19] Mahon R., Murphy A. & Lanigan W. Terahertz holographic image reconstruction and analysis (IEEE, New York, 2004).

[b20] Cherkassky V. S. *et al.* Terahertz imaging and holography with a high-power free electron laser (IEEE, New York, 2005).

[b21] Heimbeck M. S., Kim M. K., Gregory D. A. & Everitt H. O. Terahertz digital holography using angular spectrum and dual wavelength reconstruction methods. Opt. Express 19, 9192–9200 (2011). 21643173 10.1364/OE.19.009192

[b22] Ding S., Li Q., Li Y. & Wang Q. Continuous-wave terahertz digital holography by use of a pyroelectric array camera. Opt. Lett. 36, 1993–1995 (2011). 21633426 10.1364/OL.36.001993

[b23] Xue K., Li Q., Li Y. & Wang Q. Continuous-wave terahertz in-line digital holography. Opt. Lett. 37, 3228–3230 (2012). 22859141 10.1364/OL.37.003228

[b24] Gabor D. Microscopy by reconstructed wave-fronts. Proc. Roy. Soc. A 197, 454–487 (1949).

[b25] Latychevskaia T. & Fink H. W. Solution to the twin image problem in holography. Phys. Rev. Lett. 98, 233901 (2007). 17677906 10.1103/PhysRevLett.98.233901

[b26] Latychevskaia T. & Fink H. W. Simultaneous reconstruction of phase and amplitude contrast from a single holographic record. Opt. Express 17, 10697–10705 (2009). 19550466 10.1364/oe.17.010697

[b27] Guizar-Sicairos M., Thurman S. T. & Fienup J. R. Efficient subpixel image registration algorithms. Opt. Lett. 33, 156–158 (2008). 18197224 10.1364/ol.33.000156

[b28] Latychevskaia T., Longchamp J. N. & Fink H. W. When holography meets coherent diffraction imaging. Opt. Express 20, 28871–28892 (2012). 23263128 10.1364/OE.20.028871

[b29] Latychevskaia T. & Fink H. W. Resolution enhancement in digital holography by self-extrapolation of holograms. Opt. Express 21, 7726–7733 (2013). 23546153 10.1364/OE.21.007726

[b30] Latychevskaia T. & Fink H. W. Coherent microscopy at resolution beyond diffraction limit using post-experimental data extrapolation. Appl. Phys. Lett. 103, 204105 (2013).

[b31] Fienup J. R. Phase retrieval algorithms: a comparison. Appl. Opt. 21, 2758–2769 (1982). 20396114 10.1364/AO.21.002758

[b32] Goodman J. W. Introduction to Fourier Optics. (Roberts & Company Publishers, Colorado, 2004).

[b33] Hack E. & Zolliker P. Terahertz holography for imaging amplitude and phase objects. Opt. Express 22, 16079–16086 (2014). 24977861 10.1364/OE.22.016079

